# A Novel Method for Evaluating Mobile Apps (App Rating Inventory): Development Study

**DOI:** 10.2196/32643

**Published:** 2022-04-15

**Authors:** Rachel Mackey, Ann Gleason, Robert Ciulla

**Affiliations:** 1 Connected Health Branch Defense Health Agency Tacoma, WA United States

**Keywords:** mobile health apps, app rating, app analysis methodology, app market research, mobile phone

## Abstract

**Background:**

Selecting and integrating health-related apps into patient care is impeded by the absence of objective guidelines for identifying high-quality apps from the many thousands now available.

**Objective:**

This study aimed to evaluate the App Rating Inventory, which was developed by the Defense Health Agency’s Connected Health branch, to support clinical decisions regarding app selection and evaluate medical and behavioral apps.

**Methods:**

To enhance the tool’s performance, eliminate item redundancy, reduce scoring system subjectivity, and ensure a broad application of App Rating Inventory–derived results, inventory development included 3 rounds of validation testing and 2 trial periods conducted over a 6-month interval. The development focused on content validity testing, dimensionality (ie, whether the tool’s criteria performed as operationalized), factor and commonality analysis, and interrater reliability (reliability scores improved from 0.62 to 0.95 over the course of development).

**Results:**

The development phase culminated in a review of 248 apps for a total of 6944 data points and a final 28-item, 3-category app rating system. The App Rating Inventory produces scores for the following three categories: evidence (6 items), content (11 items), and customizability (11 items). The final (fourth) metric is the total score, which constitutes the sum of the 3 categories. All 28 items are weighted equally; no item is considered more (or less) important than any other item. As the scoring system is binary (either the app contains the feature or it does not), the ratings’ results are not dependent on a rater’s nuanced assessments.

**Conclusions:**

Using predetermined search criteria, app ratings begin with an environmental scan of the App Store and Google Play. This first step in market research funnels hundreds of apps in a given disease category down to a manageable top 10 apps that are, thereafter, rated using the App Rating Inventory. The category and final scores derived from the rating system inform the clinician about whether an app is evidence informed and easy to use. Although a rating allows a clinician to make focused decisions about app selection in a context where thousands of apps are available, clinicians must weigh the following factors before integrating apps into a treatment plan: clinical presentation, patient engagement and preferences, available resources, and technology expertise.

## Introduction

### Background

The lack of guidelines for identifying high-quality apps from the overwhelming number of available apps creates confusion, forestalling clinical adoption. A 2019 Australian study by Byambasuren et al [1] found that two-thirds of general practitioners used mobile apps professionally but used them primarily for medical reference purposes. In the report by Byambasuren et al [1], barriers to using apps to supplement patient care included a knowledge deficiency regarding effective uses and concerns that access sources were not trustworthy. A recent review of psychological health apps noted that it falls to health care providers to evaluate the apps’ literature and marketplace or follow the guidance of their colleagues or the health system [2]. The lack of guidelines and the time it takes to vet apps to find those most suited for clinical presentation have the potential to deter clinicians from integrating mobile apps into patient care and clinical practice.

Beyond a description of the app, user ratings, and testimonials, app distribution platforms neither describe an app’s overall quality nor indicate whether an app can meet a clinician’s needs. Descriptions posted on app stores by the software developer may be inconsistent with the app’s actual content. User ratings may imply a consensus concerning an app’s usability but do not necessarily reflect an app’s evidence or accuracy [3]. Although a popular app may be easy to use, and usability and navigability are important considerations, it may lack therapeutic value [4].

User ratings are only moderately correlated with objective rating scales and may reflect only limited experience with an app’s capabilities [5]. Pointing to the need for standardized measures, Powell et al [6] noted that someone searching for an app in the app store is likely to select the first one noticed. User ratings’ ineffectiveness in qualifying an app as medically appropriate or safe has contributed to a call for a criteria-based approach that would allow for a more objective appraisal [4]. The absence of a standardized means for evaluating health apps may impede the potential for health apps to be adopted, as well as have the potential to impact patient outcomes [7]. According to Neary and Schueller [5], evaluating apps using a structured rating tool can provide users with systematic and objective information to support the informed use of such technologies.

### Existing App Rating Systems

Rating guidelines are features or characteristics to consider when determining an app’s viability and fit for clinical practice [5]. The authors noted the value of multidimensional ratings over those with a singular focus. Baumel et al [4] included the following components: classification (eg, intended audience), usability (eg, ease of use), visual design, user engagement (eg, an app’s interactive properties), content, therapeutic persuasiveness and alliance (therapeutic rationale and relatability), a subjective evaluation of the app, credibility, and privacy and security. Password protection and the ability to import and export data, whether using the app carries potential risks, and whether technical support is available are additional considerations [6].

Oyebode et al [8] listed personalization, self-monitoring, reminders, surface credibility, social support, trustworthiness, expertise, and real-world feel as some of the most important features of health apps. Persuasive design is defined as the use of technology to change users’ attitudes or behaviors and includes behavior reinforcement, behavior change, and the shaping of attitudes as success measures [9]. Of note, although having ≥1 persuasive feature is effective, incorporating too many persuasive features only increases complexity, making the app less user-friendly and possibly decreasing its effectiveness [8].

Other factors that facilitate app use include easy-to-use navigation, clear layouts and designs, and visually available health data trends, whereas barriers can be both app specific (onerous or unintuitive navigation and small font size) and user specific (lack of technology literacy, negative attitudes about technology, and lack of internet connectivity) [10]. Jeffrey et al [10] also noted the importance of educational features and customization to support user adoption and the positive impact on adoption if the app is recommended by the patient’s practitioner.

A rapid review of the literature and web resources on app rating systems has revealed several standardized approaches to rating apps. Predominantly, these solutions help users select quality apps by providing a list of evaluation questions to be considered before using an app from the Google Play or Apple Store platforms. Comprehensive rating models provided by the Mobile App Rating Scale and PsyberGuide can be used to assess the usability of a mobile app [11] and assess the evidence supporting an app’s content [5]. The Enlight system was developed after an extensive literature review of app rating methods. Enlight seeks to specifically evaluate the therapeutic value of health apps [4]. Other notable systems include those developed by the American Psychiatric Association (APA) [12], the Anxiety and Depression Association of America [13], and the UK National Health Service [14]. The National Health Service hosts a web-based library of approved apps, the Anxiety and Depression Association of America posts ratings of selected mental health apps on the web, and the APA has a comprehensive system for rating mental health apps that is accessible from their website. In addition, several researchers have published app rating models for specific topics. The App Quality Evaluation Tool nutrition app rating system is an example of a topical rating system [15]. The app rating models, as well as a brief description of each system, are summarized in Table 1.

**Table 1:** App rating systems.

Inventory or organization	Type and total items	Availability	Intended audience
ADAA^a^-reviewed mental health apps	Apps reviewed by mental health professionals based on 5 categories	Available on the ADAA website under "mobile apps"	Mental health professionals
App adviser: APA^b^	Comprehensive app evaluation model: 5 categories with 7 to 9 questions each; brief version has 8 questions total	Available on the APA [12] website	Mental health professionals
AQEL^c^	Rating scale; 51 items	Web-based questionnaire referenced in DiFilippo et al [15]	Nutrition professionals
Enlight	Research-based, comprehensive app rating system with 6 categories of rankings from very poor to very good	Tool shared in the Baumel et al [4] publication	Health professionals
HITAM^d^	Identifies factors that influence app users’ acceptance of technology and behavior, such as health information seeking, social networking, and interactivity	See Kim and Park [16] for more information	App developers
MARS^e^	Professional app quality rating scale; 6 sections with 29 items; user scale has 26 items	Tool shared in the Stoyanov et al [11] publication	Health professionals
NHS^f^	App ratings conducted by experts and posted on the website	Available on the NHS digital website under "NHS Apps Library"	General audience
One Mind PsyberGuide	App ratings conducted by experts and posted on the website	Available on the One Mind PsyberGuide website	Mental health professionals

^a^ADAA: Anxiety and Depression Association of America.

^b^APA: American Psychiatric Association.

^c^AQEL: App Quality Evaluation Tool.

^d^HITAM: Health Information Technology Acceptance Model.

^e^MARS: Mobile App Rating Scale.

^f^NHS: National Health Service.

### Setting the Stage for the App Rating Inventory

The Defense Health Agency’s Connected Health branch is home to the research team that developed the App Rating Inventory. This branch serves as a technology resource for the Military Health System (MHS), receiving requests for mobile apps’ information from providers and app developers. A standardized approach for mobile health (mHealth) market research and app evaluation is required to ensure that consistent and reliable information is provided to MHS clinicians. The research team worked with other app evaluation teams to determine whether a pre-existing tool could be modified to fit MHS needs; however, it was determined that existing tools did not meet the criteria required for use within the MHS.

To support the MHS mission, an app evaluation tool must be usable for the full spectrum of medical and behavioral conditions and be valid for use with civilian and government-developed apps. A critical requirement was that the rating system avoid subjectively defined scoring items; what was needed was an objective tool with clear and concise criteria free from personal opinion. Of equal importance was the need for a holistic accounting of each evaluated app; that is, the rating tool should include aspects that have been tested and vetted more than evidence, user experience, the value of the content; however, all 3 should be within one system. Following a review of the literature and the existing rating systems, the research team found that no existing tool met its needs.

Although disease-specific apps are the primary use case, the App Rating Inventory can also be used with nonclinical conditions (eg, activity counters, nutrition, and physical fitness). In addition to assisting clinicians from diverse disciplines with app selection, the tool is used in decision-making concerning new software development proposals and scanning the markets for similar products before committing research funds to new development.

## Methods

### App Selection Procedure

**Figure 1:**
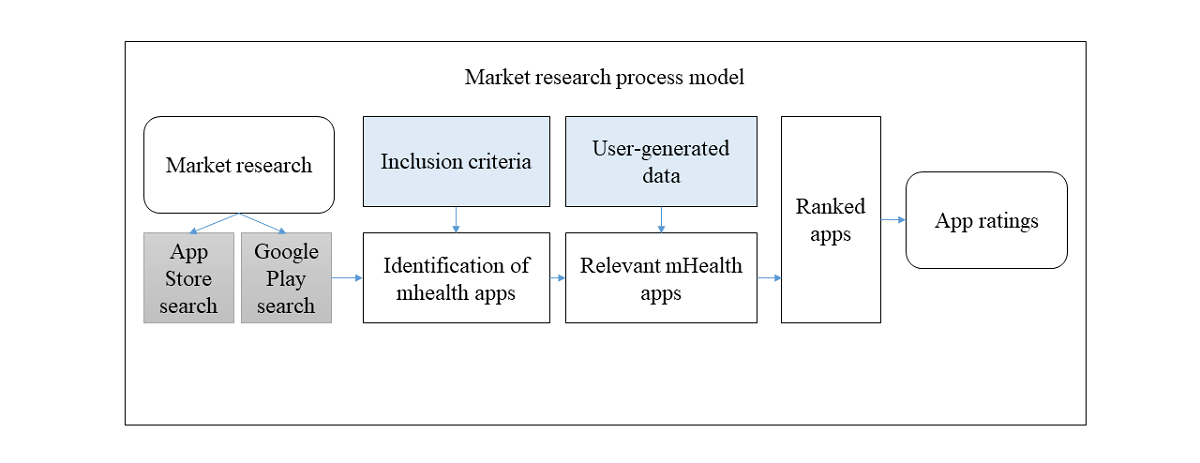
App rating process flowchart. mHealth: mobile health.

Although a decision regarding an app’s best fit for a clinical situation is supported and perhaps driven by the ratings’ findings, app selections are ultimately grounded in clinical judgment. The first step in this iterative procedure is market research. In this procedure, a market search of the distribution platforms is performed before the App Rating Inventory rating system is applied. The initial market scan leads to a more detailed review of each app and its published description before alignment with the inclusion criteria, or a decision about which apps will be rated can be determined. The protocol integrates the components of each research question. Apps that meet the inclusion criteria are funneled based on the number of criteria met. If >10 apps meet the inclusion criteria, a top-10 list is created using user-generated data from the app distribution platforms: number of user reviews, user ratings, and number of downloads, followed by the actual ratings. The process methodology for market research leading to app ratings is shown in Figure 1.

Early in the development process, an ANOVA was conducted to determine whether the above-described ranking process was statistically valid. The research team selected 10 top-ranked, 5 middle-ranked, and 10 bottom-ranked apps for testing with ANOVA. This was done to determine the level of variance between high- and low-ranked apps. Each of the 25 apps was rated using the App Rating Inventory, and the resulting scores were evaluated. Apps in the top-ranked grouping averaged an App Rating Inventory score of 14.77 (SD 4.63). The mean score for the middle-ranked apps was 9.66 (SD 2.25). Bottom- or lower-ranked apps received a mean App Rating Inventory score of 7.28 (SD 3.6). The ANOVA showed a statistically significant difference in the rating scores from top-ranked apps when compared with middle-ranked and bottom-ranked apps. No significant difference was observed between the middle-ranked and bottom-ranked apps. In short, the use of user-generated data to perform app rankings was found to be an effective method for selecting apps to be rated.

### Development of the App Rating Inventory

Following a review of the literature and existing rating systems, it was determined that a pre-existing tool did not meet the needed requirements for use within the Defense Health Agency. An app rating tool was needed that could be used by the research team to objectively assess the quality and features of all mHealth apps, regardless of specialty. The seven subject matter experts who comprised the research team created a baseline list of the characteristics that high-quality mobile apps, which are intended for use in a clinical setting, should have. The following list was based on experience and insights from information technology staff, app developers, mHealth content experts, health care providers, and health research professionals: (1) empirical base (underlying theoretical model), (2) educational content, (3) patient-generated data, (4) interactive features, (5) entertaining and immersive, (6) user customization, (7) ease of use, and (8) free of bugs and glitches.

These eight categories served as an initial baseline upon which to build the rating system. Ongoing refinement, which was focused on operationalizing terms and eliminating ambiguity and overlap between items, produced several distinct iterations. Each new format of the inventory was piloted, tested, and subjected to an in-depth review before making additional modifications. The initial 40-item inventory was subsequently reduced to its current 28-item count.

## Results

### Overview

In the initial development of the App Rating Inventory, 3 rounds of testing were performed to narrow the criteria and refine the scope of the tool. The first 2 rounds of developmental tests yielded low interrater reliability (between 0.48 and 0.50). After retraining, streamlining inventory questions, and refining operational definitions, the third round of pilot testing increased the interrater reliability score to 0.62.

Following the improvement in interrater reliability, the app development team conducted the first round of analysis, implementation, and testing of the tool for 6 months, which included 96 apps rated by the research team, and the rated apps canvassed 12 distinct conditions (eg, depression, low back pain, autism, opioid use, and stress). The 2688 data points from these ratings were used for factor and commonality analyses. Validity testing was conducted following each of the 3 pilot iterations and the subsequent revisions.

During the same 6-month period, interrater reliability (2 raters) across each of the 12 topic areas was high (between 0.92 and 0.95). The inventory’s now-improved internal consistencies allowed for more advanced testing. Commonality testing identified high levels of linkages among the 4 criteria, resulting in the deletion or combination of these criteria to reduce redundancy. Factor analysis resulted in the restructuring of the linked criteria and the removal of 2 additional criteria that were identified as outliers and did not match the features in the 96 rated apps. Content validity testing illuminated weaknesses that reduced the apps’ ability to perform well when administered to all mHealth apps, regardless of the topic area. The affected items were adapted to increase the consistency of all the apps. The App Rating Inventory proved to have effective utility across a broad range of clinical condition areas (eg, pain apps, substance abuse apps, and insomnia apps).

Statistical analysis and external consultation highlighted the following additional criteria of importance: privacy, peer support, emerging technology, and the encryption of exported data. The development team consulted 2 evidence-based published app evaluation owners identified by the preliminary literature review. Consultation with these expert sources was conducted both before the tools were created and during the development of the App Rating Inventory. The following criteria were adapted or added to bridge these gaps: the app connected users with social support (peer chat, social media, or support group platforms); the app included privacy settings and allowed encryption of user information and password protection; and the app used artificial intelligence (eg, chatbot and coach).

A second round of analysis tested the tool’s dimensionality; that is, whether the tool’s criteria were performing as operationalized. This analysis tested the predominant themes and linkages in the tool. Reliability testing was used to assess internal and external consistency. Internal analyses included rater impressions during the tool’s use and tracking of the consistencies of information across research topics. Interrater reliability was evaluated throughout the testing process.

Dimensionality testing confirmed that the tool’s hypothesized criteria performed as desired. Each of the tool’s components reflected a unique measure. Reliability testing demonstrated that the tool performed consistently. Consistencies in ratings involving apps with disparate features and across various topics (eg, pain and insomnia) showed the tool’s capacity for broad-spectrum application.

### App Rating Inventory in its Final Iteration

The final App Rating Inventory was a 28-item, 3-criterion tool (see Multimedia Appendix 1 for the App Rating Inventory checklist). The scoring system changed with each iteration of the inventory, with the end goal being a more simplified procedure. In the final version, the evidence criterion contained 6 items, and the content and customizability criterion each contained 11 items. Scoring is based on a simple binary system; that is, either the app contains the feature, or it does not. The 28 items are weighted equally; no item is considered more (or less) important than any other item. Each rated app receives four scores: a score for evidence, content, and customizability and a total score (sum of the 3 categories). Higher scores indicate that the app obtained a positive score on more items than a similar app with a lower score. Evidence, content, and customizability scores allow clinicians to make focused decisions when selecting an app for clinical use. As the prevailing assumption is that clinical judgment supersedes ratings, a clinician might select an app that receives an overall lower total score than that of similar apps, as the app received a high customizability rating that especially fits the clinician’s use case.

A binary approach means that raters do not have to grade their assessment along a continuum such as the systems reported in the literature that use a multipoint Likert-type scale. Using scoring for presence (rated as *1*) versus absence (rated as *0*), the App Rating Inventory minimizes subjective rater input. Although there may be value in developing broad constructs that apply to a full range of health technology platforms (*eHealth intervention programs*) [6], the App Rating Inventory’s sole focus is mobile apps. A system developed to measure a mobile app’s properties is not likely to equate to a website or telehealth platform.

### Case Example

To search for apps that help with sleep difficulties, the distribution platforms were queried for *sleep* and *insomnia*. Extraneous results were removed from the initial findings of 1005 apps, leaving 487 (48.46%) apps. These apps were further funneled to include only apps that were free and patient focused and included sleep education; mindfulness or meditation; fatigue or risk assessment; components of cognitive behavioral therapy; and tracking to monitor sleep quantity, quality, or impact of insomnia. The final count was 8 apps that met ≥4 criteria; these apps were rated using the App Rating Inventory.

Table 2 shows the scores for 2 sleep-related apps from the overall search (in this example, both apps were developed by the federal government). There are category and total scores (the sum of the numerators for each category). Generally, only apps that achieve at least a 50% agreement threshold (scoring positively on at least 14 items) are included in the final narrative report. In the final report, detailed descriptions of the apps are accompanied by numerical ratings. The report might also include *first-hand* observations that occurred to the rater and a gap analysis when the distribution platforms did not offer apps that met the prescan inclusion criteria, especially helpful when determining whether to fund new software development.

**Table 2:** App Rating Inventory app rating scores.

Apps	Total App Rating Inventory score	Evidence, score out of N	Content, score out of N	Customizability, score out of N
CBT-i Coach	19	6/6	7/11	6/11
Insomnia Coach	19	5/6	7/11	7/11

## Discussion

### Lessons Learned

After 3 years of consistent use of the App Rating Inventory, the development team arrived at 6 fundamental observations, as discussed in the following sections.

#### Popularity Does Matter

Apps with a high number of downloads and user reviews (suggesting that the app is popular with users) may actually reflect app quality. Although total downloads do not ensure that an app is evidence based or has clinical utility, a high number of user reviews and associated positive ratings are signs of tangible and sustained user engagement that suggest that an app has updated, relevant quality features. In the absence of consensus resources for evaluating mHealth apps, users will choose apps with high user ratings, similar to picking one restaurant over another as it has a better star rating and later finding that it does indeed have quality food, ambience, and customer experience.

#### Dynamic, Interactive Content Creates Repeat Users

For apps developers, increasing app engagement is an important consideration. The repeated use of dynamic content by self-management and prevention-focused apps will increase the number of touchpoints a patient has with the associated content. These engaging features range from app reminders, pushed as notifications to the user’s main device home screen, to dynamic, adaptable content that evolves as the user meets individual app goals. In the end, there is a feedback loop between app sustainment and a loyal following—loyalty incentivizes the developer to improve the app, and those improvements are rewarded by more loyalty.

#### The Bait and Switch Method is Common

With mobile apps, what you see is not always what you receive; in fact, there is no equivalence between distribution platforms’ descriptions and what a reader can expect from a research article’s abstract. The description of an app is similar to a sales pitch meant to encourage downloads. Once downloaded, the user experience may not match the marketing ploy. Perhaps, the most common occurrence is supposedly free content that the user discovers has a cost, or the user may find that the key content is locked in the free version. The user may discover that a subscription package is required; the common tagline for this is *free to download*. Indeed, the app is free to download but, once installed, cannot be used without selecting a monthly purchase plan. Although a medication management app may allow the user to enter the medication that they want to track, access to the symptom tracker, medication reminders, patient diary, calendar, and refill reminders are all separate in-app purchases or only available if the user chooses to purchase a *premium* version of the app.

#### There Really is an App for Everything

Most smartphone users will be familiar with the phrase “there’s an app for that.” From 2015 to 2017, the number of public-facing mHealth apps doubled, saturating distribution platforms with >300,000 apps [17]. The market is flooded with mHealth apps for almost every aspect of care and health. When conducting original app market research, it is common to have several hundred apps generated by each search term, regardless of the topic area. The most time-intensive step of the app rating process is the initial environmental scan, identifying alignment with inclusion criteria and determining an app’s topical relevance.

#### Apps Can Perpetuate Inaccurate Information

App distribution platforms do not require mHealth apps to be evidence informed or supported by best practices. As apps may contain inaccurate or potentially harmful content, vetting and validating an app’s clinical content before recommending it is crucial.

#### Mobile Apps Can Enhance the Patient Experience

The integration of mHealth apps into care has been shown to increase treatment fidelity and program adherence [18]. By increasing patient touchpoints, health literacy, and health efficacy, mHealth apps can meet the patient at where they are.

### Key Considerations

The decision to recommend apps in clinical settings should be based on a comprehensive algorithm that presents diagnosis, technology literacy, app quality and content, treatment planning, accessibility and cost, and data security. Critically underlying this decision matrix is clinical judgment. Deciding which app to use may also depend on patient engagement; a low level of engagement suggests that the app should be primarily educational, whereas an app oriented toward behavior change might be more suitable for highly engaged patients [19]. Clearly, patient characteristics factor into determining an app’s suitability for use in treatment or self-management [20,21].

Although a patient’s input should be obtained along with the clinician’s assessment of the app [3], a commentary (in the literature) that a qualified appraisal of an app’s value should be grounded in the therapeutic alliance and not solely based on an objective scoring system begs the following question: are the two mutually exclusive? Powell et al [6] suggested the notion that a scoring system gets a clinician started with app selection; however, before recommending it, the responsible clinician would download and explore all of the app’s features regardless of how well it scores on an objective scoring system.

The multistep app vetting process proposed by Boudreaux et al [3] includes a literature review, a search of clearinghouses and app stores, a review of app descriptions and user ratings, reviewing social media entries (both professional and patient networks), piloting the app, and obtaining feedback from patients. The case study cited in the 2014 Boudreaux et al [3] article begins with a hypothetical physician who is interested in celiac disease apps. First, the physician contacts a medical librarian to search for a systematic review. This first step in the series of aforementioned steps raises the following question: how many clinicians will have access to or sufficient free time to check in with a medical librarian? More to the point, the absence of randomized controlled trials that a literature review would reveal does not mean that useful celiac apps are not available. The physician’s next steps were as follows: searching a clearinghouse, looking at user ratings in iTunes, and then taping web-based social networks, finally leading to the physician selecting an app—at which point the physician uses the app for a day and then recommends it to a patient. These steps have value but are time intensive and, in the end, fail to obtain an independently determined, objective evaluation of the app’s content. A busy clinician needs quick, digestible guidance regarding an app’s merits and usefulness.

Rating systems require some initial investment in learning the scoring protocol and the system’s theoretical basis. Although the App Rating Inventory research team strived to develop a system that minimized the focus on esthetic measures (potentially introducing a degree of subjectivity into the rating system), the App Rating Inventory scoring nevertheless requires initial training to best understand the meaning of the inventory’s 28 items. Although the amount of time to complete a rating depends on the number and complexity of features contained within the app, experienced raters can complete an App Rating Inventory app rating in between 15 and 40 minutes.

As the App Rating Inventory was designed for use across medical conditions and with apps developed by both government and commercial vendors, the scoring system can be used outside of the MHS and by nongovernment research groups. However, it should be noted that although any clinician can use the App Rating Inventory, the inventory was developed principally for the research team’s use, with the rating results reported directly to MHS providers. Although using the entire App Rating Inventory is the recommended use case, it is possible to evaluate only an app’s evidence, content, or customizability depending on the clinician’s needs. This type of targeted use would produce an individual score for only 1 or 2 constructs of interest. Importantly, familiarity with the App Rating Inventory can help clinicians gain insight into the components that go into a well-constructed mobile app.

However, some writers in this space argue that rating approaches that produce a score are flawed. Henson et al [20] discussed a consensus statement concerning mobile app standards, noting that a key feature of the framework (privacy and security, app effectiveness, user experience, and data integration) is that none of the framework questions are associated with a score. This concern with a system that produces an objective score is that such a score has the potential to be reductionist or somehow imply the existence of a magic formula [22]. Although there is a certain logic in the APA’s stepwise hierarchical process, a clinician who has the time to categorically navigate the APA system would surely conclude that 1 app was better than similar apps. After all, the clinician must choose which among an array of apps to include in the treatment plan. It is unclear how this inevitable ranking is significantly different from the end result of an objective rating or decision matrix.

Another argument against a scoring system is that software developers are constantly making changes to apps. Excluding bug fixes, what is the evidence that developers are making constant upgrades to an app? Even bug fixes, although making an app more usable, do not necessarily alter the core content or graphics. Only a wholesale upgrade that results in an entirely new graphical interface or a navigation system renovation or removing or adding entirely new features or content would negate the results from an objective scoring system. Although content should be systematically monitored by subject matter experts involved in the app’s development, what is the rate of occurrence of new medical or behavioral knowledge that would necessitate significant changes to an app’s features and navigation? Consider the following for behavioral treatment apps: how often do new theoretical models emerge that would substantially alter an app intended to help with depression, stress management, or insomnia?

Perhaps, mobile apps should be subject to a certification system [6]. Although there have been attempts to certify software companies as meeting standards, there are currently no well-trusted or actively used certifications for individual apps. Although Google and Apple check for security issues, apps posted on these distribution platforms are not subjected to a certification evaluation. A centralized, curated, and easily accessed database might list both certified and uncertified apps. However, even with agreement across the industry about how a quality app is defined, it is unlikely that even the most comprehensive library will include the full spectrum of health-specific apps. Other considerations include the following: should clinicians only use apps listed in the clearinghouse; would ratings be considered obsolete after a year, necessitating a new round of evaluation; who would track updates that significantly alter the app’s content; If a patient brings a noncurated app into an appointment, should the clinician discourage its use even if the patient reports that the app is beneficial; how would interrater reliability be accomplished to ensure certification accuracy; and who is the final arbiter of the app’s guidelines and the associated ratings?

Selecting a best-practice app should involve no more than the following three steps: (1) query the market with key search terms; (2) check the description, user ratings, total downloads, and credibility of the developer; and (3) download and navigate the app with a particular focus on whether the content is evidence based, is founded in a theoretical model, and allows the user to input and store information (interactivity).

Should a viable clearinghouse exist, clinicians might avoid the first 2 steps; however, assuming that no clearinghouse is comprehensive, the last step is crucial. Even when the professional rating of an app is available, the last step is an essential requirement.

In summary, scoring systems provide guidance and filter down an exhaustive list of health apps in a given category to a handful for consideration. Indeed, apps are not new medicines; in many cases, they are novel delivery systems for proven interventions.

## Appendix

Multimedia Appendix 1 App Rating Inventory checklist.

## Abbreviations

APA: American Psychiatric Association
mHealth: mobile health
MHS: Military Health System
