# Supplementary material for: A Novel Method for Evaluating Mobile Apps (App Rating Inventory): Development Study
Source: JMIR Mhealth Uhealth. 2022 Apr 15;10(4):e32643. doi: 10.2196/32643 (PMC9055478; doi:10.2196/32643)
Supplement: Multimedia Appendix 1 [file mhealth_v10i4e32643_app1.pdf]

App Rating Inventory Checklist

EVIDENCE

Medical/ Behavioral Focus:

(a) App content focuses on a behavioral or medical concern;
(b) Content reflects actual diagnostic nomenclature for the indicated problem;
(c) Content is consistent with clinical recommendations/ practice guidelines.

Direct/ Indirect Evidence:

(a) The app itself was involved in at least one formal study (such as a randomized controlled trial);
(b) There is reference either in the app store or within the app, or can be ascertained by a subject expert, that the app's content is based on the empirical literature.

Theoretical Model:

(a) The app's content/ interventions are based on an empirically-validated treatment model, such as cognitive-behavioral therapy.

CONTENT

Treatment-Focus:

(a) The app provides information regarding types of treatment(s) that are commonly administered to reduce or prevent the problem's occurrence;
(b) The app identifies treatments that lack empirical support and should be avoided;
(c) The app provides skills-learning features to help ameliorate symptoms (such as a deep-breathing exercise to manage stress or low-back exercises to manage pain).

User-Generated Data:

(a) The user can input responses to questionnaires/ self-assessments;
(b) The user can input information into behavioral tracking systems (moods, activities, nutrition, glucose measures, etc.);
(c) The user can input situational triggers/ characteristics concomitant with medical/ behavioral conditions;
(d) The app prompts the user to input specific characteristics (age, gender, height, weight, etc.) and provides the user with tailored information;
(e) The app can sync with user personal contacts' information
(f) User-inputted information is storable and available from one use session to the next

External Links:

(a) The app contains links/ recommendations to educational information, aid hotlines, and/or additional referral resources.
(b) The app connects the user with social support (peer chat, social media, and/or support group platforms).

CUSTOMIZABILITY

Editing:

(a) User-generated data can be systematically edited, removed/ disabled, cleared, or re-set, to include personal contact information, self-report scales, calendars, reminders/ notifications, start-up tips, and settings;

(b) The app includes privacy settings and allows the encryption of user information and/or password protection.
(c) Multimedia can be customized (graphical backgrounds can be varied, background music selections can be selected from an array, audio can be paused/ turned on/off).

Engagement:
(a) The user receives extrinsic rewards during use of the app in response to achieving user-defined or app-specified milestones (gamification);
(b) The app allows the user to integrate information within the app with the user's actual, surrounding environment (augmented reality);
(c) The app utilizes artificial intelligence (AI chat bot, AI coach, etc.)

Ease-of-Use:
(a) There is a main menu on the home screen;
(b) The app provides text-based and/or video-demonstrated start-up instructions;
(c) There is a help function that indexes and troubleshoots often-asked how-to questions;
(d) There is an FAQ feature;
(e) The app's buttons are organized, readable, and of sufficient size that touching a button on any given screen does not inadvertently launch an adjacent button and, when activated, the button leads to expected results.
